# MAPPING COLLABORATION RELATIONSHIPS IN AGE-FRIENDLY COMMUNITIES INITIATIVES

**DOI:** 10.1093/geroni/igz038.3364

**Published:** 2019-11-08

**Authors:** Althea Pestine-Stevens

**Affiliations:** School of Social Work, Rutgers University, New Brunswick, United States

## Abstract

Age-friendly initiatives (AFIs) convene stakeholders throughout a community to improve social and built environments for long lives. Despite rapid growth in AFIs worldwide, research on how AFIs operate, sustain, and impact their communities has been slow to develop. This poster presents a new social network analysis (SNA) survey instrument, which can be used to advance research on AFIs by identifying key relationships and activities that drive collaborative community change processes. The survey asks a representative from each organizational member of an AFI coalition to select “partner” organizations with whom they have worked on AFI goals. Respondents then select from a list of activities in which they engage with each partnering organization. The questions regarding collaboration activities were developed based on theories of inter-sectoral and community-wide collaboration, SNA studies of collaboration in health prevention networks, and qualitative interviews with leaders of an established AFI coalition in Upstate New York. This tool was administered with respondents from 18 organizations comprising the New York coalition. Administration of the pilot indicated that the questions were acceptable and feasible for participants to complete. Analysis of the data through SNA software (UCINET) yielded visual maps to understand dimensions of the AFI’s inter-organizational network. Local government offices and nonprofits emerged as central network nodes for connecting stakeholders. Findings also indicated denser networks around lower-intensity collaboration activities, such as sharing information, relative to higher-intensity activities, such as sharing financial resources. Implications of the tool for future research on the development of AFIs across diverse community contexts are discussed.

